# No evidence for a role of MHC class II genotype in the chemical encoding of heterozygosity and relatedness in Antarctic fur seals

**DOI:** 10.1098/rspb.2023.2519

**Published:** 2024-03-20

**Authors:** Jonas Tebbe, Katja Havenstein, Jaume Forcada, Ralph Tiedemann, Barbara Caspers, Joseph I. Hoffman

**Affiliations:** ^1^ Department of Evolutionary Population Genetics, Faculty of Biology, Bielefeld University, 33615 Bielefeld, Germany; ^2^ Department of Behavioural Ecology, Bielefeld University, 33501 Bielefeld, Germany; ^3^ Department of Animal Behaviour, Bielefeld University, 33501 Bielefeld, Germany; ^4^ Unit of Evolutionary Biology / Systematic Zoology, Institute of Biochemistry and Biology, University of Potsdam, Potsdam-Golm, Germany; ^5^ British Antarctic Survey, High Cross, Madingley Road, Cambridge CB3 OET, UK; ^6^ Joint Institute for Individualisation in a Changing Environment (JICE), Bielefeld University and University of Münster, Bielefeld, Germany; ^7^ Center for Biotechnology (CeBiTec), Faculty of Biology, Bielefeld University, 33615 Bielefeld, Germany

**Keywords:** odour, major histocompatibility complex, heterozygosity, relatedness, Antarctic fur seal (*Arctocephalus gazella*), pinniped

## Abstract

Despite decades of research, surprisingly little is known about the mechanism(s) by which an individual's genotype is encoded in odour. Many studies have focused on the role of the major histocompatibility complex (MHC) owing to its importance for survival and mate choice. However, the salience of MHC-mediated odours compared to chemicals influenced by the rest of the genome remains unclear, especially in wild populations where it is challenging to quantify and control for the effects of the genomic background. We addressed this issue in Antarctic fur seals by analysing skin swabs together with full-length MHC DQB II exon 2 sequences and data from 41 genome-wide distributed microsatellites. We did not find any effects of MHC relatedness on chemical similarity and there was also no relationship between MHC heterozygosity and chemical diversity. However, multilocus heterozygosity showed a significant positive association with chemical diversity, even after controlling for MHC heterozygosity. Our results appear to rule out a dominant role of the MHC in the chemical encoding of genetic information in a wild vertebrate population and highlight the need for genome-wide approaches to elucidate the mechanism(s) and specific genes underlying genotype-odour associations.

## 1. Introduction

Chemical communication is the oldest and most primitive means of information transfer and underpins many aspects of vertebrate social life, from mate attraction and partner choice through parent-offspring communication to kin recognition [1]. In vertebrates, body odours are complex mixtures of chemicals that are produced either directly for the purpose of olfactory signalling (e.g. secretions of specific scent glands) or indirectly as by-products of metabolic processes [1]. Thus, body odours can act as multidimensional, individual-specific signals that encode diverse information such as sex, age, health, reproductive status and even genotype [2,3].

There is clear evidence from many vertebrate species that individuals are capable of recognizing kin using olfactory cues alone [4–7]. The fact that surface chemicals can even be used to identify unfamiliar kin [8] points to the existence not only of individual-specific odours, but also to a covariation between genetic relatedness and olfactory similarity [9]. By implication, genome-wide heterozygosity should also be reflected in an individual's odour. Much of the variation in genome-wide heterozygosity among individuals within a population occurs owing to inbreeding, i.e. the mating of close relatives, which increases the proportion of the genome that is identical by descent [10]. However, ‘surprisingly little progress' has been made in understanding the link between genotype and odour in vertebrates [11]. This is partly owing to the difficulty of disentangling genetic versus environmental sources of variation [12] but also because the small panels of around 10 microsatellite loci typical of most studies are often underpowered to detect genetic associations [12] as they provide imprecise estimates of genome-wide heterozygosity (i.e. inbreeding) and relatedness [13].

A major focus of previous studies of the genetic mechanisms underlying olfactory kin recognition has been the major histocompatibility complex (MHC), an extraordinarily diverse cluster of genes that plays a central role in pathogen detection and antigen binding [14,15]. MHC heterozygosity, particularly at the MHC class II genes, often confers a fitness advantage [16,17] by increasing resistance to parasites and pathogens that would otherwise negatively impact key life-history traits such as growth and reproductive success [18–20]. Thus, in order to optimize MHC diversity, many vertebrate species have evolved MHC disassortative mating strategies mediated by MHC-based olfactory recognition mechanisms [14,15]. In line with this, the ‘MHC peptide hypothesis’ argues that specific peptide ligands directly encode olfactory information about MHC genotype [21]. This is supported by several studies that have detected MHC peptides in mouse and human urine [22,23]. Elsewhere, it has been argued that the MHC might influence odour indirectly via the microbiome [24,25] although evidence in support of this hypothesis remains limited [25,26].

Regardless of the exact mechanism by which MHC genotype is chemically encoded, the MHC plays a central role in chemical communication in many species [24,27–32]. Nevertheless, the importance of MHC-mediated odours compared to chemicals influenced by the rest of the genome remains unclear [33–35]. Furthermore, to be useful in the wild, olfactory markers of genetic traits ‘must be recognizable against the variable genetic and environmental background of normal outbred populations' [11, p. 3]. Hence studies are needed that analyse the role of the MHC in the context of the genomic background, for example by quantifying and controlling for the effects of background genes [35].

A further complication is that much of our current knowledge about the mechanisms underpinning vertebrate olfactory communication comes from studies of model organisms such as mice [9,34,35]. Most of these studies have been conducted in the laboratory, where the availability of congenic animals and MHC mutants has allowed the effects of the MHC to be separated from the genomic background [22,36,37]. Furthermore, behavioural experiments and controlled crosses can be performed in a laboratory setting, which allow precise manipulation of an individual's genotype and the evaluation of behavioural outcomes such as individual recognition and MHC-dependent mate choice [22,36–38]. However, despite these important advantages, laboratory studies of the role of the MHC in odour production and mate choice have not entirely escaped criticism as the use of inbred lineages that differ only at the MHC might introduce experimental artefacts [35]. For example, Wyatt [35] argued that females might choose MHC dissimilar partners simply because the rest of the genome is invariant. Moreover, genetic drift within inbred lines might in some cases have led to the accumulation of small genetic differences in non-MHC regions that may influence odour [34]. For these and other reasons, more naturalistic studies involving outbred populations of non-model species are desirable, but it is even more challenging under these circumstances to quantify and control for the potentially confounding effects of the genomic background [34].

Pinnipeds are well suited to studying chemical communication as they possess large repertoires of functional olfactory receptor genes [39] and are sensitive to even the faintest of odours [40]. Fur seals in particular have a strong musky smell [41] that has been attributed to facial glands which hypertrophy during the breeding season [42,43], and naso-nasal inspection plays an important role in pup recognition [44,45], suggesting that olfactory communication plays an important role in fur seals during the peak reproductive period. In Antarctic fur seals (*Arctocephalus gazella*), chemical fingerprints (otherwise commonly referred to as ‘odour profiles’, ‘scent profiles’ or ‘semiochemical profiles’) robustly and repeatably encode various information that could be important for kin recognition, including colony membership and mother-offspring similarity [12,46]. Furthermore, heterozygosity is known to be positively associated with chemical diversity, defined as the total number of substances in an individual's chemical fingerprint [12]. However, the potential involvement of the MHC in the chemical encoding genetic information in fur seals remains to be investigated.

Here, we addressed this question by combining previously published chemical data and 41-locus microsatellite genotypes from Stoffel *et al*. [12] with MHC class II DQB exon 2 sequences [47]. Given that MHC class II genes have been implicated in survival and disease susceptibility in pinnipeds [48–50] and the MHC class II DQB exon 2 shows evidence of balancing selection and trans-species polymorphism in Antarctic fur seals [47], we hypothesized that this locus may be involved in the chemical encoding of genetic information in this species. Specifically, we hypothesized that (i) individuals with more similar MHC genotypes should be more similar in their chemical fingerprints; (ii) MHC heterozygotes should have higher chemical diversity than MHC homozygotes; and (iii) these hypothesized associations might only become apparent after controlling for the potentially confounding effects of relatedness and genome-wide heterozygosity respectively. We furthermore hypothesized that (iv) variation in chemical fingerprints among individuals may be associated with the possession of different MHC genotypes and/or alleles.

## 2. Methods

### (a) Field methods

**Figure 1.**
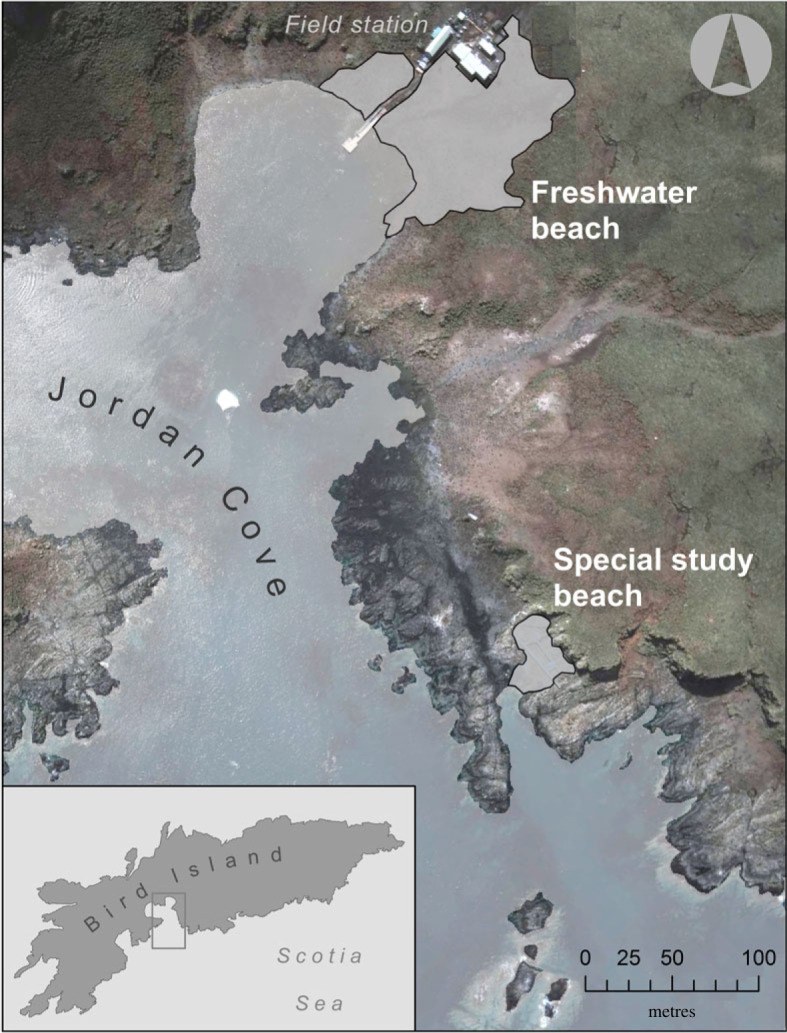
Map of Bird Island, South Georgia showing the geographical locations (grey) of two Antarctic fur seal breeding colonies, the Freshwater beach and Special study beach.

Chemical and genetic samples were analysed from a total of 88 Antarctic fur seals (44 mother-offspring pairs) during the 2011/2012 field season on Bird Island, South Georgia (54°00′24.8″ S, 38°03′04.1″ W). Sampling was carried out at two breeding colonies (figure 1): ‘Freshwater beach’ (FWB) and the ‘Special study beach’ (SSB), which are separated by around 200 m [51]. Chemical samples were collected by rubbing the cheeks of the animals with sterile cotton wool swabs and stored individually in 60% ethanol at –20°C. Tissue samples were taken from the foreflipper using piglet ear notching pliers and stored at –20°C in 20% dimethyl sulphoxide saturated with salt [52].

### (b) Chemical analysis

The chemical samples were subjected to gas chromatography–mass spectrometry (GC-MS) as described by Stoffel *et al*. [12]. The resulting chemical data were then quality checked and corrected for minor retention shifts using ‘GCalignR’ as described by Ottensmann *et al*. [53]. After log(x + 1) transforming the chemical data, we used ‘vegan’ [54] to calculate pairwise chemical dissimilarity using Bray–Curtis values, which were then converted to pairwise chemical similarity values by subtracting them from one. Chemical diversity was calculated as the total number of substances in each individual's chemical fingerprint [12].

### (c) Genetic analysis

Total genomic DNA was extracted and genotyped at 43 microsatellites (41 of which conformed to Hardy–Weinberg equilibrium and were retained for analysis) as described by Stoffel *et al*. [12]. Individual standardized multilocus heterozygosity (sMLH) and pairwise relatedness were quantified using ‘inbreedR’ [55] and ‘Demerelate’ [56], respectively. We also used inbreedR to calculate *g*_2_, a measure of the extent to which heterozygosity is correlated across loci, and to derive its 95% confidence interval through 1000 permutations of the microsatellite dataset. Subsequently, intronic primers were designed to polymerase chain reaction (PCR) amplify the full-length second exon of the MHC DQB II locus, which was multiply cloned and sequenced in each individual as described by Tebbe *et al*. [47]. From an initial sample set of 88 individuals, PCR products were obtained for 56 individuals, resulting in a dataset of 771 full-length MHC DQB II exon 2 sequences (mean = 13.8, range = 7–32 sequences per individual). Some of these samples could not be successfully sequenced owing to small amounts of DNA being available, low yields of PCR products despite multiple attempts, and the cloning not producing interpretable sequences for all of the samples. As the MHC DQB II exon 2 locus appears not to be duplicated and conforms to Mendelian inheritance in Antarctic fur seals [47], we calculated MHC heterozygosity (1 = heterozygous, 0 = homozygous) and used ‘phyloseq’ [57] to quantify pairwise MHC relatedness as 1 - UniFrac distance. UniFrac distances allowed us to incorporate phylogenetic distances of the MHC DQB exon 2 alleles based on an already existing tree [47] by calculating fractions of unshared branch lengths between pairs.

### (d) Statistical analysis

To evaluate the contribution of the MHC towards the chemical encoding of genetic information in Antarctic fur seals, we fitted multiple linear mixed effect models in R [58]. Specifically, we used a multi-model framework to test for the effects of genetic predictor variables on chemical similarity and chemical diversity, respectively. The implemented models are shown below:

(i) model a1: chemical similarity ∼ MHC relatedness + colony membership + age + (1|family) + (1|pair ID1) + (1|pair ID2);

(ii) model a2: chemical similarity ∼ microsatellite relatedness + colony membership + age + (1|family) + (1|pair ID1) + (1|pair ID2);

(iii) model a3: chemical similarity ∼ MHC relatedness + microsatellite relatedness + colony membership + age + (1|family) + (1|pair ID1) + (1|pair ID2);

(iv) model a4: chemical similarity ∼ colony membership + age + (1|family) + (1|pair ID1) + (1|pair ID2);

(v) model b1: chemical diversity ∼ MHC heterozygosity + colony membership + age + (1|family);

(vi) model b2: chemical diversity ∼ sMLH + colony membership + age + (1|family);

(vii) model b3: chemical diversity ∼ MHC heterozygosity + sMLH + colony membership + age + (1|family); and

(viii) model b4: chemical diversity ∼colony membership + age + (1|family).

For the models of chemical similarity, colony membership was a fixed effect with two levels (same versus different colony), age was a fixed effect with two levels (same versus different age class) and family was a random effect with two levels (same versus different family). The identities of both individuals being compared were also included as random effects (pair ID1 and pair ID2, respectively). For the models of chemical diversity, colony membership and age were similarly fitted as fixed effects with two levels (FWB versus SSB and mother versus pup, respectively). We also included family ID as a random effect in these models. Although we controlled for age in the above models, we also repeated these models separately for mothers and pups, excluding age and family as covariates.

We used the R package ‘performance’ [59] to identify the best performing models. Performance uses a weighted approach that incorporates multiple ranking criteria (Akaike information criterion (AIC), Akaike information criterion corrected for small sample size, Bayesian information criterion, conditional and marginal *r*^2^, intraclass correlation coefficient, root mean square error, *σ*) in the model selection procedure while including random effects. As an additional check, we also used the ‘dredge’ function in ‘MuMln’ [60] in conjunction with partial *r*² values of each combination of fixed effects, calculated with ‘partR2’ [61]. However, this approach only uses AIC for model selection and it also excludes random effects. The quality of model fit was also assessed using residual and quantile-quantile plots.

After identifying the best performing models of chemical similarity and chemical diversity, we implemented bootstrap analyses to evaluate the uncertainty associated with our effect size estimates. Specifically, we randomly selected 1000 bootstrap samples of 800 pairwise values for the chemical similarity model and 30 individual values for the chemical diversity model and then calculated partial *r*² values to extract the effect sizes of MHC relatedness, microsatellite relatedness, MHC heterozygosity and sMLH.

Next, we used a stepwise decomposition of Bray–Curtis similarities (SIMPER) to identify a subset of chemicals that maximized chemical similarity between all pairs of individuals in the dataset. SIMPER calculates similarity percentage values that reflect the contribution of each chemical towards overall pairwise Bray–Curtis similarity. After visual inspection of the cumulative curve depicting the percentage similarity contributions of the chemicals from the highest to lowest contributions, we selected a subset of 15 chemicals that maximized the slope while explaining over 35% of the total variation. We then re-ran the models of chemical similarity described above to test for an association between MHC relatedness and chemical similarity at this subset of chemicals.

Finally, we tested for chemical differences between mothers and pups, between the two colonies, among different MHC genotypes and in relation to the presence/absence of specific MHC alleles using non-parametric permutational multivariate analyses of variance (PERMANOVA). For non-normally distributed pairwise data, this approach tests for within- and among-group differences in the centroids and dispersion of pre-defined groups, in our case based on Bray–Curtis similarity values. Statistical significance was computed based on 9999 permutations of the data. All analyses were performed using a combination of standard functions in vegan and custom scripts in R.

## 3. Results

To test for the possible involvement of the MHC in the chemical encoding of genetic information in Antarctic fur seals, we combined GC-MS and microsatellite data [12] with MHC class II DQB exon 2 sequences [47] from 56 individuals, comprising 32 mothers and 24 pups. The number of chemicals per individual averaged over the entire dataset was 55.9 ± 2.8 (electronic supplementary material, table S1) and no significant differences in chemical diversity were found between mothers and pups or between the two colonies (ANOVA, age: *F*_1,52_ = 0.13, *p* = 0.72; colony: *F*_1,52_ = 0.27, *p* = 0.609; age*colony: *F*_1,52_ = 3.46, *p* = 0.068). However, testing for group differences with PERMANOVA, we found that chemical similarity was significantly affected by an individual's colony of origin (pseudo-*F*_1,1_ = 11.47, *p* = 0.001) and by family (pseudo-*F*_1,34_ = 1.73, *p* = 0.001), but not by age class (Pseudo-*F*_1,55_ = 1.09, *p* = 0.330). The two-locus heterozygosity disequilibrium estimator was positive and significant (*g*_2_ = 0.0039, 95% CI = 0.005–0.0073, *p* = 0.008), indicating that the 41 microsatellites capture genome-wide variation in inbreeding. We identified a total of 19 MHC alleles and 37 distinct MHC genotypes. Relatedness at the MHC was weakly but significantly associated with microsatellite relatedness (*F*_1,923_ = 4.55, *p* = 0.033) but no association was found between MHC heterozygosity and sMLH (*F*_1,52_ = 1.45, *p* = 0.233).

### (a) Model selection

**Figure 2.**
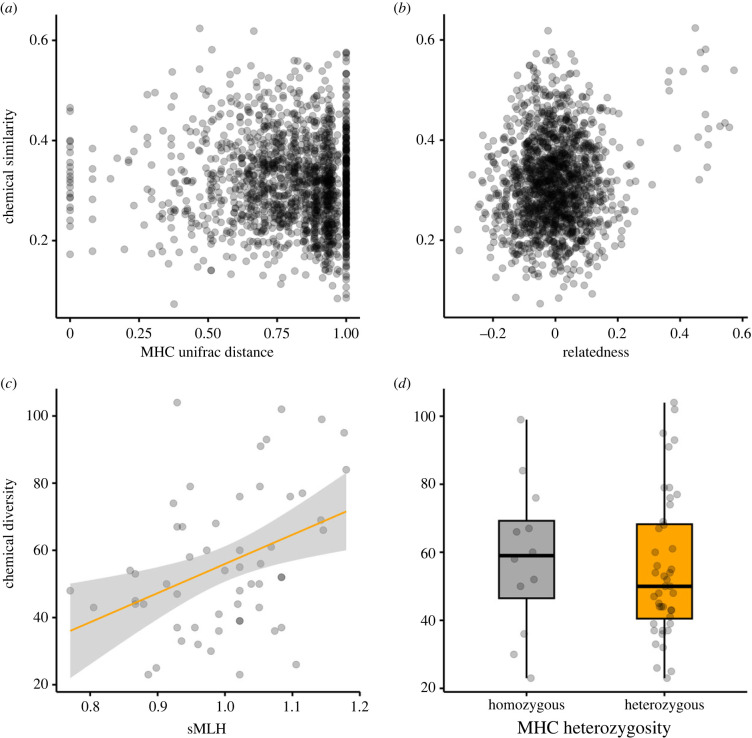
Relationship between genetic variation at the MHC and microsatellites and chemical variation in Antarctic fur seals. (*a*) The relationship between pairwise MHC UniFrac distance and chemical similarity (model a1, not significant). (*b*) The relationship between genetic relatedness at 41 microsatellite loci and chemical similarity (model a2, not significant). (*c*) The relationship between microsatellite heterozygosity (sMLH) and chemical diversity (model b2, *F*_1,44_ = 4.55, *p* = 0.003). (*d*) Differences in chemical diversity (defined as the number of chemicals in an individual's chemical fingerprint) between MHC homozygotes and MHC heterozygotes (model b1, not significant). The box plots show the median and interquartile range, with the whiskers indicating the 95% confidence intervals (CIs). Linear regressions and associated 95% CIs are only shown for the statistically significant relationship between chemical diversity and sMLH.

We used a model comparison approach implemented in the ‘performance’ package to identify the best performing models of chemical similarity and chemical diversity based on a weighted comparison of multiple model performance measures (see Methods for details). When all of the animals were analysed together, the best model of chemical similarity did not include any main genetic effects (model a4; electronic supplementary material, table S2a). Specifically, chemical similarity was not significantly associated with neither MHC relatedness (figure 2*a*) nor microsatellite relatedness (figure 2*b*). The best performing (null) model only included colony and age as fixed effects, as previously found by Stoffel *et al*. [12] and Tebbe *et al*. [46]. The best model of chemical diversity (model b2; table 1*b*) included only sMLH as a fixed genetic effect (*p* = 0.003; figure 2*c*; electronic supplementary material, table S2b) while no significant effects of MHC heterozygosity on chemical diversity were found (figure 2*d*). Similar results were obtained when the mothers and pups were analysed separately (electronic supplementary material, tables S3 and S4).

**Table 1.** The relative performance of alternative models of (*a*) chemical similarity; and (*b*) chemical diversity in Antarctic fur seals (see Methods for details). (These models differed only in their fixed effects, while the random effects remained the same, as described in the Methods. Multiple model performance criteria are presented together with overall performance scores calculated with the ‘compare_performance’ function in performance [40]. The models with the best overall performance are highlighted with asterisks. *r*^2^ (cond.), conditional *r*^2^; *r*^2^ (marg.), marginal *r*^2^; ICC, intraclass correlation coefficient; RMSE, root-mean-square-error; *σ*, residual standard deviation; AIC, Aikake information criterion; BIC, Bayesian information criterion.)

model	main genetic effects	*r*^2^ (cond.)	*r*^2^ (marg.)	ICC	RMSE	sigma	AIC weights	AICc weights	BIC weights	performance score (%)
(*a*) chemical similarity										
a1	MHC relatedness	0.691	0.137	0.642	0.060	0.062	0.199	0.198	0.025	48.06
a2	relatedness	0.686	0.140	0.635	0.060	0.062	0.208	0.207	0.027	28.70
a3	MHC relatedness + relatedness	0.684	0.141	0.633	0.060	0.062	0.081	0.079	0.000	12.50
a4*	none	0.693	0.137	0.644	0.060	0.062	0.512	0.515	0.947	87.50
(*b*) chemical diversity										
b1	MHC heterozygosity	0.741	0.001	0.741	7.295	10.842	0.006	0.006	0.007	6.84
b2*	sMLH	0.768	0.115	0.738	6.731	9.987	0.710	0.754	0.830	90.84
b3	MHC heterozygosity + sMLH	0.746	0.113	0.735	6.742	10.117	0.269	0.220	0.114	56.44
b4	none	0.746	0.001	0.746	7.249	10.668	0.015	0.020	0.050	19.44

As an additional check, we also used the ‘dredge’ function of the MuMln package for model selection. In support of the results described above, the best model of chemical similarity contained only colony as a fixed effect (electronic supplementary material, table S5). The best performing model of chemical diversity contained not only sMLH but also MHC heterozygosity as fixed genetic effects (electronic supplementary material, table S6). However, the goodness of fit of this model was virtually identical to that of a model containing only sMLH as a fixed effect (partial *r*^2^ = 0.1168 versus 0.1166, electronic supplementary material, table S6). This difference is marginal bearing in mind that dredge only uses a single performance criterion for model selection and does not account for random effects.

**Figure 3.**
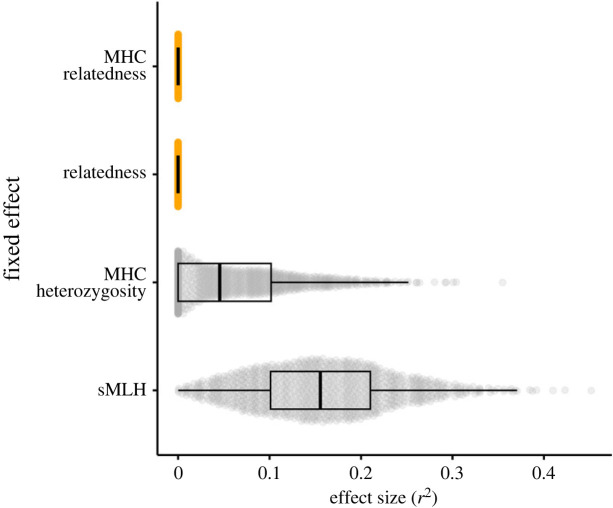
Bootstrapped partial *r*^2^ values for the fixed effects of the models. The plot shows 1000 bootstrapped data points for each of the four main genetic effects evaluated in the models—MHC relatedness, microsatellite relatedness, sMLH and MHC heterozygosity. The box plots show the medians and interquartile ranges, with the whiskers indicating the 95% confidence intervals.

To quantify the uncertainty in our effect size estimates, we used a bootstrapping approach as described in the Methods. For the best supported model of chemical similarity, the effect size distributions of both MHC relatedness and microsatellite relatedness were tightly centred around zero (figure 3), providing further evidence for a lack of any obvious genetic effects on chemical similarity. For the best supported model of chemical diversity, the 95% CI of the effect size distribution of MHC heterozygosity also overlapped zero, although there was a tail of positive bootstrapped *r*^2^ values, whereas sMLH had a clearly positive effect size distribution centred around an *r*^2^ value of approximately 0.16 with a 95% CI that did not overlap zero.

### (b) Controlling for the genomic background

To test for any effects of the MHC that might be masked by the genomic background, we compared the model of chemical similarity containing only MHC relatedness as a main genetic effect (model a1) with the equivalent model containing both MHC relatedness and microsatellite relatedness (model a3). In neither model was a significant effect of MHC relatedness found (electronic supplementary material, table S2a). Similarly, we compared models of chemical diversity containing only MHC heterozygosity (model b1) and both MHC heterozygosity and sMLH (model b3). Again, no significant effects of the MHC were detected in either model (electronic supplementary material, table S2b) although reassuringly, the effect of sMLH remained statistically significant (*p* = 0.003) even after controlling for MHC heterozygosity.

### (c) Testing for major histocompatibility complex effects on specific chemicals

In order to decrease the complexity of our data and to minimize the noise contributed by large numbers of chemicals that do not substantially contribute towards overall chemical similarity, we used a stepwise decomposition of Bray–Curtis similarities (SIMPER). This approach allowed us to identify a specific subset of substances that contributed the most towards the total variation in chemical similarity between all pairs of individuals in the dataset. A cumulative plot of the proportional contribution of each chemical towards Bray–Curtis similarity identified 15 substances that together accounted for over 35% of the variation in chemical similarity (electronic supplementary material, figure S2). Repeating the model of chemical similarity based only on these substances produced virtually identical results to those described above (electronic supplementary material, tables S7 and S8).

### (d) Testing for genotype- and allele-specific effects of the major histocompatibility complex

Finally, we tested for more subtle effects of the MHC on chemical fingerprints using a PERMANOVA framework. First, we tested for chemical differences among the 37 different MHC genotypes. We found that MHC genotype did not explain a significant proportion of the total chemical variation (pseudo-*F*_1,36_ = 1.08, *p* = 0.17; electronic supplementary material, figure S1). Second, we tested for chemical differences among individuals in relation to the presence/absence of the 19 different MHC alleles. Again, we could not detect any significant effects after table-wide false discovery rate correction for multiple tests (electronic supplementary material, table S9).

## 4. Discussion

Owing to its central role in fitness and mate choice, the MHC has been a major focus of studies of chemical communication in vertebrates [14,15]. However, interpreting associations between MHC genotype and odour is not always straightforward, especially given that few studies are able to quantify and control for the genomic background, especially in natural populations of non-model organisms [33–35]. Here, we investigated the role of the MHC in encoding chemical similarity and diversity in a wild population of Antarctic fur seals. Using a multi-model statistical framework, we found that the best model of chemical similarity did not include any genetic effects, while the best model of chemical diversity included only multilocus heterozygosity. Our findings suggest that information about an individual's genotype may be chemically encoded in fur seals via mechanism(s) that do not involve the MHC.

Mate choice experiments have shown that many species, including humans, are able to discriminate the body odours of individuals based on their MHC genotype [62,63]. However, relatively little is known about the mechanism(s) by which MHC genes influence individual odours. One possibility is that MHC genotype has a direct influence on odour owing to the presence of degradation products of MHC molecules and/or secondary metabolites of biochemical pathways modulated by the MHC [38,64]. Alternatively, MHC genotype might indirectly influence odour by mediating the interaction between the host immune system and odour-producing bacteria [24,33]. The former mechanism predicts a positive relationship between MHC similarity and chemical similarity, as well as higher chemical diversity in MHC heterozygotes compared to MHC homozygotes. By contrast, a negative relationship between MHC heterozygosity and chemical diversity might be expected if MHC heterozygotes were better able to suppress bacterial diversity [65].

Our results are not consistent with either of these mechanisms, as we were unable to detect any effects of the MHC on chemical similarity or diversity, regardless of whether we tested for relationships in isolation or after controlling for the genomic background. Broadly speaking, our results stand in contrast to several recent studies of wild vertebrate populations reporting links between the MHC and odour [66–70]. However, in support of our results, Slade *et al*. [68] also found no relationship between MHC heterozygosity and the chemical diversity of preen wax in song sparrows, even though chemical distance was associated with MHC dissimilarity in male-female but not same sex dyads. Furthermore, Setchell *et al*. [67] did not find odour differences among mandrills carrying different MHC supertypes, although MHC similarity and chemical similarity were correlated. Moreover, associations between the MHC and odour often appear to be context-dependent, varying for example by sex or breeding status [66,68,69]. However, there is no indication of strong context-dependence in our study as comparable results were obtained for breeding females and offspring.

There is a growing appreciation of the need to incorporate the potentially confounding effects of the genomic background into MHC studies, especially in natural populations [33–35]. For example, MHC heterozygosity can correlate with genome-wide heterozygosity when there is inbreeding within a population [71], which could potentially lead to effects of MHC heterozygosity being confounded by inbreeding and *vice versa*. However, it is challenging to incorporate the effects of the genomic background into studies of wild populations, where genomic resources are often lacking and characterizing and sequencing the MHC already requires a considerable investment of time and resources. Fortunately, a two-decade long genetic study of Antarctic fur seals [72] has provided us with the tools to genotype a sufficiently large number of microsatellites to capture a clear signal of identity disequilibrium, which gives us a reasonable degree of confidence that MHC heterozygosity and inbreeding are uncorrelated. This lack of association is arguably to be expected given that the MHC DQB II exon 2 shows evidence of being under balancing selection in Antarctic fur seals [47], while diversity across the rest of the genome is known to be strongly influenced in this species by genetic drift during a historical bottleneck [73–75] and inbreeding depression [72,76–78] These contrasting selective pressures could result in patterns of diversity at the MHC becoming dissociated from patterns of diversity across the rest of the genome.

The MHC did not feature in any of the top models of chemical similarity or chemical diversity, no effects of the MHC were found after controlling for the genomic background, and no chemical associations were found with specific MHC genotypes or alleles. However, we focussed on broad patterns so cannot rule out the possibility of one or a small number of chemicals being affected by the MHC. Nevertheless, our results are clearly at odds with previous studies reporting systemic effects of the MHC on odour [67–70]. By contrast, we found a highly significant positive relationship between heterozygosity and chemical diversity, as previously reported by Stoffel *et al.* [12], which suggests the inbreeding is encoded in Antarctic fur seal chemical fingerprints. Consequently, the lack of any obvious parallel effects of the MHC points towards one or more, as yet unknown mechanism(s), being involved in the chemical encoding of inbreeding.

How might inbreeding be chemically encoded if not via the MHC? We can envisage a number of possibilities. Chiefly among them, several studies have shown that inbreeding alters the expression of tens to hundreds of genes, including those involved in metabolism, stress responses, inflammation and immunity [79,80]. These alterations to an organism's physiology might result in systematic chemical changes that could alter patterns of chemical diversity. Alternatively, inbreeding might indirectly influence chemical diversity by changing microbial alpha diversity. In line with this, we have previously shown that heterozygous fur seals carry less diverse skin microbiota [65]. This is consistent with the leash model of host control, which argues that better quality individuals should be more effective at keeping their microbiome ‘on a leash’ [81]. In the context of Antarctic fur seals, a negative relationship between heterozygosity and microbial diversity would then argue for outbred individuals being more capable of suppressing non-beneficial microbes, possibly owing to higher levels of immunogenetic diversity [65]. However, it is unclear how a reduction in microbial diversity could lead to increased chemical diversity.

One potential criticism of our study is that we focused on a single MHC class II locus and did not investigate the possible involvement of MHC class I genes. However, the MHC DQB II exon 2 is the most obvious candidate to investigate in Antarctic fur seals for three reasons. First, MHC class II genes are involved in the presentation of extracellular pathogens such as bacteria to the immune system [82]. Given that host microbial communities have been implicated in the generation of host odours via the fermentation hypothesis [83,84], a focus on MHC class II genes therefore seems justified. Second, the MHC DQB II exon 2 genotype has previously been linked to offspring survival [48] and habitat specific selection [85] in a closely related pinniped species, the grey seal. Third, the MHC DQB II exon 2 is known to be expressed in Antarctic fur seals [86] and the putative antigen binding sites encoded by this locus show patterns of amino acid divergence consistent with balancing selection and trans-species polymorphism [47]. Nevertheless, our MHC haplotypes are only 267 base pairs long and they do not span multiple MHC loci, which would be desirable for a more complete characterization of the effects of the MHC on odour. While this represents an interesting potential future research avenue, gathering long-range haplotype information is non-trivial in wild populations owing to the technical difficulty and cost of sequencing and phasing multiple loci in non-model organisms.

There is also the question of whether our sample size of 56 individuals is sufficiently large to detect effects of MHC genotype on odour. To interpret the results of tests with non-significant results, biologists often advocate *post hoc* power analyses. However, the approach of calculating the observed power given the value of a test statistic is flawed because there is a one to one relationship between the *p*-value and the power to reject the null hypothesis of no effect [87–90]. A better alternative is to present effect sizes together with their 95% CIs, which are influenced by both sample size and the variance of the data, and which therefore provide an indication of the likelihood of the true effect size being zero [87–90]. Bootstrapping our dataset over individuals revealed that the 95% CIs of the effect size distributions of MHC relatedness and microsatellite relatedness on chemical similarity were both tightly centred around zero. The 95% CI of the effect size distribution of MHC heterozygosity on chemical diversity also overlapped zero, although a minority of the bootstrapped datasets produced positive *r*^2^ values. By contrast, the bootstrapped effect size distribution of the relationship between sMLH and chemical diversity was clearly positive with a 95% CI that did not overlap zero. These results allow us to conclude with a reasonably high degree of confidence that there is no association between MHC relatedness and chemical similarity in our dataset. However, we prefer to be more circumspect about the lack of a relationship between MHC heterozygosity and chemical diversity, where a larger sample size of individuals (and in particular the inclusion of more MHC homozygotes) would improve the resolution of the effect size distribution. However, a simple comparison of the empirical effect sizes of MHC heterozygosity and sMLH suggests that, if present, any effect of MHC heterozygosity on chemical diversity would probably be orders of magnitude smaller than the effect of sMLH, which again argues for a genome-wide effect.

Another potential criticism of our study is that behavioural variation, especially in the amount of time animals spend at sea, might potentially confound our results. However, Antarctic fur seal mothers give birth and provision their pups ashore for several days before they first leave on a foraging trip [91] and they are also site faithful both within and between seasons [92]. The pups also move very little during the first 20 days of life [93] and their natal coat lacks the water-repellent properties of adult fur, preventing them from spending prolonged periods of time at sea [94]. Hence, behavioural variation in the context of our study is rather limited. In line with this, sMLH shows a strong association with chemical diversity and, more generally, chemical patterns in Antarctic fur seals are highly repeatable among years and across different contexts [46] which would not be expected if there were confounding effects of individual variation in behaviour.

Finally, although we deployed substantially more microsatellites than is usually possible, genomic approaches such as reduced representation sequencing would undoubtedly enhance the resolution of inbreeding [95]. Nonetheless, our genetic dataset carries a clear signature of identity disequilibrium, indicating that 41 microsatellites is adequate to capture genome-wide variation in inbreeding in Antarctic fur seals. We also found a significant positive relationship between sMLH and chemical diversity, implying that outbred individuals possess more diverse chemical fingerprints. Overall, our genetic resolution is therefore sufficient to detect strong, genome-wide relationships, although future studies using higher resolution approaches based on larger numbers of single nucleotide polymorphisms would represent a marked improvement by allowing genome-wide scans for loci or genomic regions that influence animal odours.

In summary, our study lends further support to existing arguments for broadening the focus of chemical studies beyond the MHC [33–35]. In particular, although it may be pragmatic to focus on specific MHC genes, our limited understanding of the broader context, both in relation to other MHC loci and to the rest of the genome, could be problematic. We would like to see the next generation of chemical studies incorporating approaches capable of screening larger numbers of genome-wide distributed loci. This is becoming increasingly feasible thanks to the development of cost-effective genotyping approaches such as reduced representation sequencing, the increasing availability of single nucleotide polymorphism arrays, and the falling costs of whole genome resequencing. An alternative and complementary approach would be to link patterns of gene expression to chemical differences among individuals in order to identify candidate genes whose expression levels correlate with chemical diversity and/or similarity. In principle, these and related approaches should even allow us to go beyond searching for broad associations to identifying the specific genes responsible for chemical differences among individuals.

## Data Availability

Microsatellite, MHC class II DQB exon 2 genotypes and chemical fingerprint alignments can be accessed via Zenodo: https://doi.org/10.5281/zenodo.7741581 [96]. Sequences of the 19 MHC alleles are available on GenBank (accession numbers ON060886–ON060904). The code used to analyse the data and accompanying documentation are available as a PDF file written in Rmarkdown (electronic supplementary material, file S1 [96] or https://github.com/tebbej/arga_chem_encoding). Supplementary material is available online [97].
